# Relationships among discrimination, cognitive-affective pain amplifiers, and identification with Native American culture: results from the Oklahoma Study of Native American Pain Risk

**DOI:** 10.3389/fpsyt.2025.1568450

**Published:** 2025-09-30

**Authors:** Brandon W. Jones, Hayden M. Ventresca, Taylor V. Brown, Parker A. Kell, Kayla N. Trevino, Joanna O. Shadlow, Jamie L. Rhudy

**Affiliations:** ^1^ Tobacco Settlement Endowment Trust (TSET) Health Promotion Research Center, The University of Oklahoma Health Sciences, Tulsa, OK, United States; ^2^ Department of Psychology, The University of Tulsa, Tulsa, OK, United States; ^3^ Department of Psychology, Oklahoma State University, Tulsa, OK, United States; ^4^ Department of Health Promotion Sciences, The University of Oklahoma Health Sciences, Tulsa, OK, United States

**Keywords:** ethnic differences, Native Americans, interpersonal discrimination, pain catastrophizing, pain anxiety, cultural identification

## Abstract

**Background:**

Native Americans (NAs) experience higher rates of chronic pain than other U.S. ethnic/racial groups. This may be partly caused by stress from interpersonal discrimination, which promotes pain-related catastrophizing and anxiety, cognitive-emotional processes that amplify pain. Greater identification with NA culture has been shown to buffer against negative health outcomes for NA communities, therefore the present study examined whether greater identification with NA culture buffers against the harmful effects of discrimination on pain-related anxiety and catastrophizing.

**Material and Methods:**

Participants were 153 healthy, chronic pain-free NAs enrolled in the Oklahoma Study of Native American Pain Risk (OK-SNAP). Identification with NA culture was assessed by the Native American Acculturation Scale (NAAS), which was reversed scored so that higher scores=greater identification. Interpersonal discrimination was assessed by the Everyday Discrimination Scale. Following laboratory pain tasks, situational pain catastrophizing was assessed with the Pain Catastrophizing Scale and pain-related anxiety was assessed with a visual analogue scale (VAS).

**Results:**

A significant interaction was found between discrimination and identification with NA culture when predicting pain catastrophizing (ΔR^2^ = 0.034, *p* = 0.017). Greater identification with NA culture was associated with a significant positive relationship between discrimination and pain catastrophizing (*p* < 0.001), whereas less identification was associated with a non-significant relationship (*p* = 0.32). Although discrimination was associated with higher pain-related anxiety (*p* = 0.015), this was not moderated by identification with NA culture (ΔR^2^ = 0.009, *p* = 0.23), nor did identification with NA culture predict pain-related anxiety. An additional analysis found that NAs who identified more with NA culture experienced more discrimination (*p* = 0.012).

**Conclusions:**

These findings suggest that the relationship between discrimination and pain catastrophizing is stronger for NAs who identify more strongly with NA culture, an effect that is likely due to these individuals having greater overall exposure to discrimination. Alternatively, the NAAS may not capture the full range of cultural processes that buffer against negative health outcomes for NAs. Future research should examine other facets of cultural resilience.

## 1 Introduction

Chronic pain is experienced by approximately 20% of adults in the United States and is associated with significant negative consequences (1), such as poorer occupational performance, psychological wellbeing, physical functioning, and sleep (2). Native Americans (NAs) exhibit alarmingly high chronic pain prevalence rates compared to other racial and ethnic communities in the United States – nearly 1 in 3 NAs experience chronic pain (3). The Oklahoma Study of Native American Pain Risk (OK-SNAP) found that healthy, pain-free NAs were 2.9 times more likely to develop chronic pain than non-Hispanic White (NHW) individuals within a 2-year period (4) and 4 times more likely to develop chronic pain within a 5-year period (5). This pain inequity is partly explained by experiences with interpersonal discrimination that promote pain-related anxiety and pain catastrophizing, cognitive-affective processes that amplify pain (4).

Interpersonal discrimination refers to a negative attitude or unfair treatment (e.g., harassment, exclusion, differential treatment) towards a group of people, and research has found it contributes to poorer health outcomes in underserved communities (6). NAs report experiencing interpersonal discrimination across many settings, including healthcare, police encounters, housing, and employment opportunities (7). Moreover, interpersonal discrimination is associated with higher blood pressure (8), depressive symptoms (9), and physical pain and impairment (10) for NAs. Research on non-NA populations has shown that both lifetime and daily interpersonal discrimination increases one’s likelihood of developing chronic pain (11) and our research has shown that interpersonal discrimination partially explains the higher rate of chronic pain in NAs (4, 5). Together, these findings highlight the importance of interpersonal discrimination as a potential contributor to the NA chronic pain inequity. The current manuscript aims to investigate if greater identification with NA culture buffers against the negative impact of interpersonal discrimination on pain related anxiety and pain catastrophizing.

Pain-related anxiety is a future-oriented, negative affective response to potential threats that enhances pain (12). Pain catastrophizing refers to cognitive processes involving pain magnification, rumination about pain, and feelings of helplessness, which work together to amplify the pain experience (13). Pain-related anxiety and catastrophizing are associated with lower heat pain threshold/tolerance (12, 14), heightened postoperative pain intensity (15), and increased risk for postsurgical chronic pain (16). Our previous work has shown that NAs report more pain-related anxiety and catastrophizing than NHWs in response to painful tasks (17), and these cognitive-affective pain amplifiers are associated with increased risk of prospectively developing chronic pain for NAs (5, 18, 19).

Given the detrimental impact of interpersonal discrimination and cognitive-affective pain amplifiers on chronic pain outcomes for NAs, it is important to find protective factors that ameliorate this pain disparity. Identification with NA culture is one potential factor. Indeed, Walters and Simoni introduced the Indigenist Stress Coping Model, which states that cultural processes such as identification with NA culture and spirituality are coping mechanisms for life stressors such as interpersonal discrimination and historical trauma (20). In their conceptualization, Ward & Szabó explain that cultural identification includes both enculturation and acculturation processes. Enculturation “refers to the process of being socialized into a particular culture” whereas acculturation refers to navigating the relationships between traditional and mainstream cultural identities (21, p. 268). Thus, NA cultural identification has been measured using enculturation and acculturation scales (22–24). For example, Garrett and Pichette developed the Native American Acculturation Scale (NAAS) to assess cultural identity along an acculturation continuum ranging from identification with traditional NA cultural practices to assimilated into mainstream American culture, with the midpoint representing bicultural identification (22).

Previous research has found that greater identification with NA culture is protective against alcohol use (25) and depressive symptoms (9) for NA adults. Additionally, Fetter and Thompson found that strong ethnic identity moderated (buffered) the relationship between historical loss and wellbeing in a sample of NA college students (26). Further studies with NA youth have demonstrated greater identification with NA culture is protective against suicidal ideation (27) and promotes academic success (28) and resilience (29). Moreover, a recent scoping review highlights identification with NA culture as a factor that protects against negative health outcomes because of enhanced resilience promoted by increased social support, wellbeing, and engagement with traditional practices (30). However, it has also been shown that greater identification with NA culture is associated with experiencing more interpersonal discrimination (25, 31, 32), and historical loss thinking (e.g., frequency of thinking about loss of land, people, and/or culture (25, 32);, both of which have negative health outcomes.

The current study is a secondary analysis of data collected from OK-SNAP. In OK-SNAP, healthy, pain-free NHW and NA adults were recruited to identify potential mechanisms contributing to the NA pain disparity before chronic pain had developed. Several pain tasks were administered to assess individual differences in pain sensitivity, central sensitization, and pain inhibition. However, these tasks also provided an ecologically-valid opportunity to assess pain-related anxiety and catastrophizing in response to pain – processes that amplify pain and increase chronic pain risk (33). For the present study, only data from NA participants in OK-SNAP were used, given that the study aim was to investigate whether identification with NA culture buffers against (moderates) the harmful effects of interpersonal discrimination. It was hypothesized that greater cultural identification would be associated with a weaker relationship between interpersonal discrimination and pain-related anxiety and pain catastrophizing.

## 2 Materials and methods

### 2.1 Participants

Participants included 153 healthy, pain-free NA individuals who participated in OK-SNAP. Participants were recruited from NA newspapers, fliers, personal communications with NA groups, email announcements, and online platforms (e.g., Facebook). OK-SNAP exclusion criteria were: (1) <18 years old, (2) history of cardiovascular, neuroendocrine, musculoskeletal, and neurological disorders, (3) reported chronic pain or acute pain problems, (4) BMI ≥ 35, (5) use of antidepressants, anxiolytic, analgesic, stimulant, or antihypertensive medication, (6) current psychotic symptoms or substance use problems, and/or (7) an inability to read and speak English. OK-SNAP data collection occurred from March 2014 to October 2018. Procedures were approved by the University of Tulsa, Cherokee Nation, and Indian Health Service Oklahoma City Area Office institutional review boards (IRBs). All participants provided informed consent and were able to withdraw from the study at any point. NA status was verified through Certificate of Degree of Indian Blood (CDIB) or tribal membership cards. NA participants represent tribal nations predominately from eastern Oklahoma and the southern plains, but tribal affiliation was not reported to respect tribal community confidentiality (33).

### 2.2 Procedures

OK-SNAP was performed over a 2-day testing period, lasting approximately 4 to 6 hours each testing day. A full description of study procedures is provided elsewhere (33). Screening for inclusion/exclusion criteria and informed consent were performed on the first testing day. One testing day consisted of pain sensitivity tasks including assessment of threshold and tolerance of cold, heat, ischemia, pressure, and electric pain. The other testing day assessed measures of central sensitization and pain inhibition. Central sensitization tasks consisted of nociceptive flexion response (NFR) threshold, temporal summation of NFR (TS-NFR), and temporal summation of heat pain (TS-Heat). Pain inhibition tasks included emotional controls of nociception (ECON) and conditioned pain modulation (CPM).

Situational pain catastrophizing and pain-related anxiety were assessed after each pain task. Each measure was averaged across tasks and used as the primary dependent variables in the present study. Participants were compensated $100 for each completed testing day or $10 an hour for non-completed days (33).

### 2.3 Questionnaires

#### 2.3.1 Demographics

A custom-built demographics questionnaire was used to collect background information including age at enrollment, sex assigned at birth (male vs. female), race, marital status, highest level of completed education, employment status, annual household income, and body mass index (BMI). To be included in the current study, participants were required to have chosen American Indian/Alaskan Native or mixed race, including American Indian/Alaskan Native, which was verified by presentation of a CDIB or tribal identification card. Participant height and weight were measured using a medical scale and then used to calculate BMI in kg/m^2^ (4).

#### 2.3.2 Interpersonal discrimination

The 9-item Everyday Discrimination Scale was used to assess the frequency of interpersonal discrimination experienced in daily life (34). Questions include, “You are treated with less courtesy than other people are,” “People act as if they think you are dishonest,” and “You are threatened or harassed.” Participants responded to “In your day-to-day life, how often do any of the following things happen to you?” to each of the 9 items on a 6-point Likert scale with ratings ranging from: 1 = “Almost every day,” 2 = “At least once a week,” 3 = “A few times a month,” 4 = “A few times a year,” 5 = “Less than once a year,” and 6 = “Never.” Responses were reverse scored and averaged. Thus, scores ranged from 1 to 6, with higher scores being indicative of more frequent experiences of interpersonal discrimination. A final question asked participants what they thought the main reason was for their interpersonal discrimination experiences (e.g., gender, race, age, weight, income level, sexual orientation). Cronbach’s alpha was 0.897 (4).

#### 2.3.3 Situational pain catastrophizing

The 13-item Pain Catastrophizing Scale (PCS) was used to assess situational pain catastrophizing immediately following each pain task. The PCS assesses rumination, magnification, and helplessness in response to pain. Questions include, “I anxiously want the pain to go away,” “I become afraid that the pain may get worse,” and “I worry all the time about whether the pain will end.” Participants rated each of the 13 items on a 5-point Likert scale (0 = “Not at all,” 1 = “To a slight degree,” 2 = “To a moderate degree,” 3 = “To a great degree,” and 4 = “All the time”). The 13 items of the PCS were summed to provide a total score ranging from 0-52, with higher scores indicating greater pain catastrophizing (13). To assess situational catastrophizing, instructions were modified so that participants were asked to think back to the thoughts they experienced during each pain task. The 10 total PCS scores across the 10 pain tasks had a Cronbach’s alpha of 0.92, thus an overall situational pain catastrophizing score was created by averaging across the tasks (4).

#### 2.3.4 Pain-related anxiety

A visual analog scale (VAS) was used to assess pain-related anxiety following each of the 10 pain tasks. Instructions to participants were, “Using this scale, rate how anxious the *[insert pain test]* made you feel.” Participants rated their pain-related anxiety on a scale that ranged from “not at all anxious” to “extremely anxious.” VAS responses were converted to scores ranging from 0 to 100, with higher scores representing more pain-related anxiety. Cronbach’s alpha for the 10 pain-related anxiety scores in response to the 10 pain tasks was 0.90, thus an overall pain-related anxiety score was created by averaging across the tasks (4).

#### 2.3.5 Identification with Native American culture

The 20-item Native American Acculturation Scale (NAAS) was used to assess overall identification with NA culture on an acculturation continuum (22). The NAAS includes domains related to language, identity, friendships, behaviors, generational/geographic background, and attitudes. Questions on the NAAS include “What language can you speak?,” “How do you identify yourself?,” and “What contact have you had with Native American communities?” Participants’ responses were reverse scored so that higher scores would reflect greater identification with culture to ease interpretation. Scores were then averaged creating an overall score that ranged from 1 = “lower identification with NA culture” to 5 = “greater identification with NA culture,” with a midpoint score of 3 indicating bicultural identification. Cronbach’s alpha was 0.88 (33).

### 2.4 Data analysis

Before performing primary analyses, variable distributions were inspected using box plots, histograms, and tests of normality. Outlier values were identified using the MAD-median procedure with a recommended cutoff score of 2.24 (35) and winsorized with the nearest non-outlier value. Winsorized variables in the current study were interpersonal discrimination, identification with NA culture, and situational pain catastrophizing. Age was log-10 transformed to adjust for positive skew. The following variables had missing values: interpersonal discrimination (3.9%), identification with NA culture (3.9%), income (3.3%), BMI (2.6%), marital status (2.0%), employment status (1.3%), situational pain catastrophizing (1.3%), pain-related anxiety (1.3%), and highest level of completed education (0.7%). To avoid listwise deletion in regression models, the expectation-maximization (EM) algorithm in LISREL 8.80 (Scientific Software International, Chapel Hill, NC) was performed to impute missing values. Significance level was set at α<.05 (2-tailed) for all analyses.

Two moderated regression analyses were performed using PROCESS v4.2 (36). In the first model, pain-related anxiety was the criterion variable. In the second model, situational pain catastrophizing was the criterion variable. Both models included the same predictors: interpersonal discrimination, identification with NA culture, and the interaction term between interpersonal discrimination and identification with NA culture. Primary analysis controlled for age, sex, and income given that epidemiological studies in the U.S. have found that older age, female sex, and lower socioeconomic position are chronic pain risk factors (37).

Before the creation of interaction terms, main effect variables were mean centered. Identification with NA culture was conceptualized as the moderator in both models. In the event of a significant interaction, simple effects were examined at -1SD, the mean, and +1SD of the moderator. To establish if there is a relationship between identification with NA culture and exposure to discrimination, a simple regression was conducted in SPSS v29 (IBM, Armonk, NY) with interpersonal discrimination as the criterion variable and identification with NA culture as the predictor.

## 3 Results

### 3.1 Sample characteristics

**Table 1** displays the sample characteristics. Participants’ ages ranged from 18 to 63 years, with a mean age of 30.60 years (SD = 12.67). The sample was 58.2% female. Most participants reported their marital status as single (61.4%). Most participants (78.4%) reported some college experience (partial college or college graduate). Approximately a fourth of participants reported being unemployed (25.5%). Over half of participants reported earning less than $25,000 (52.3%), which is below the average per capita income in the state of Oklahoma (38). Participants’ BMI ranged from 16.60 to 35.00, with a mean BMI of 25.88 (SD = 4.43). Approximately 15% of participants scored above a 3 on the reverse scored NAAS indicating that most (85%) participants scored in the bicultural identification or lower identification with NA culture range. Participants’ cultural identification ranged from 1.37 to 3.45. As shown in **Table 2**, 34.7% of participants reported the main reason for their interpersonal discrimination experiences were their ancestry or race. **Table 3** presents means, *SD*s, and intercorrelations among all variables used in regression models.

**Table 1:** Sample characteristics.

Categorical variables		N	%
Sex	Male	64	41.8
	Female	89	58.2
Marital Status	Single	94	61.4
	Married/coinhabiting	44	28.8
	Separated/divorced/widowed	15	9.8
Education	High school graduate or less	33	21.6
	Partial college	65	42.5
	College/graduate school graduate	55	35.9
Employment	<40 hours per week	49	32.0
	≥40 hours per week	53	34.6
	Student/retired	12	7.8
	Unemployed ≤$9,999	39 40	25.5 26.1
Income	$10,000-$14,999	20	13.1
	$15,000-$24,999	20	13.1
	$25,000-$34,999	17	11.1
	$35,000-$49,999	25	16.3
	≥$50,000	31	20.3

**Table 2:** Reported reasons for interpersonal discrimination experiences.

Participants reported main reason	N	%
Your Ancestry or National Origins	21	14.3
Your Gender	39	26.5
Your Race	30	20.4
Your Age	38	25.9
Your Religion	5	3.4
Your Height	11	7.5
Your Weight	10	6.8
Some other Aspect of Physical Appearance	35	23.8
Your Sexual Orientation	3	2
Your Education or Income Level	43	29.3

Participants were allowed to choose more than one reason for experiencing discrimination, therefore the N’s do not sum to the total sample size.

**Table 3:** Means, standard deviations, and intercorrelations for study variables.

Study Variables	M	SD	1	2	3	4	5	6	7
1.Age (years)	30.60	12.67							
2.Income	2.39	1.91	0.20^*^						
3.Sex Assigned at Birth (0 = male, 1 = female)	0.58	0.49	0.06	-0.07					
4.Interpersonal Discrimination (EDS; 1-6)	2.01	0.81	-0.02	-0.01	-0.09				
5.Identification with NA Culture (NAAS; 1-5)	2.36	0.53	0.23^*^	-0.04	0.08	0.21^*^			
6.Pain-Related Anxiety (VAS; 0-100)	44.31	21.55	0.21^*^	0.07	0.03	0.17^*^	-0.01		
7.Pain Catastrophizing (PCS; 0-42)	11.25	7.87	0.09	0.09	0.01	0.29^*^	0.01	0.71^*^	
8.Discrimination x Identification with NA Culture	0.09	0.41	-0.01	-0.17^*^	0.03	0.03	0.05	0.09	0.18^*^

To ease interpretation, all means are uncentered (except the interaction which is based on centered means). Higher reversed scores on the Native American Acculturation Scale are associated with greater identification with NA culture. EDS, Everyday Discrimination Scale.

^*^ *p* < .05 (2-tailed).

### 3.2 Predicting pain-related anxiety

**Figure 1:**
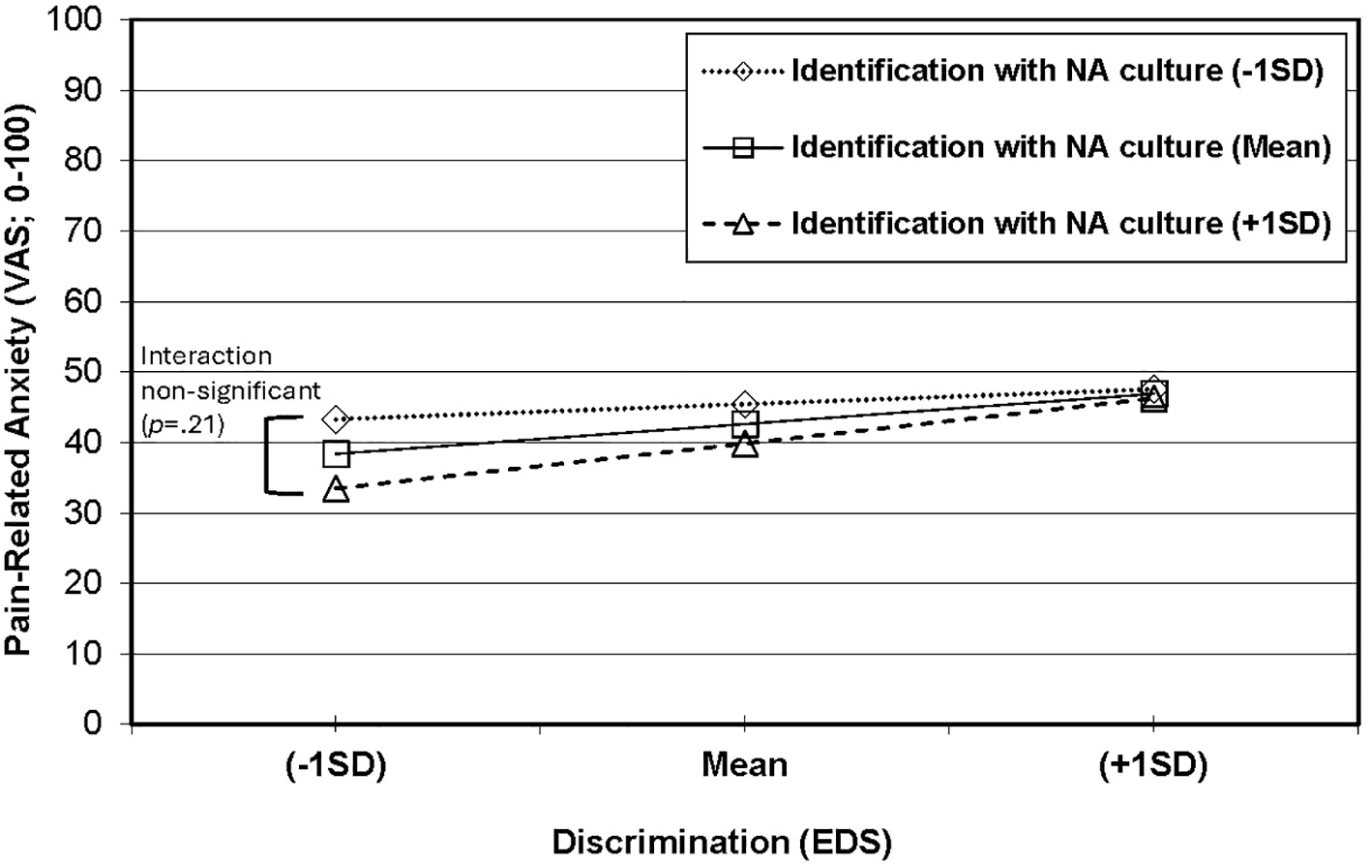
Non-significant interaction between identification with NA culture and interpersonal discrimination in the prediction of pain-related anxiety.

The overall model that included all predictors in the prediction of pain-related anxiety was significant [F(6,146) = 2.62, *p* = 0.019] and explained 9.73% of the variance. As shown in **Table 4**, there was a significant main effect for interpersonal discrimination, such that experiencing more unfair treatment was associated with experiencing higher pain-related anxiety (β = 0.20, *p* = 0.017). However, as shown in **Figure 1**, the interaction of interpersonal discrimination and identification with NA culture (higher scores=greater enculturation) was nonsignificant [F(1,146) = 1.61, *p* = 0.21] and only explained an additional 1.0% of the variance (ΔR^2^ = 0.010). There was a significant effect for age (β = 0.23, *p* = 0.005), but no other predictor was significant. These findings indicate greater experiences of interpersonal discrimination and higher age was associated with more pain-related anxiety.

**Table 4:** Moderated regression analysis predicting pain-related anxiety.

Predictor variable	*B*	*SEB*	β	*r*	*sr^2^*	Δ*R^2^*
Age (years)	31.29*	11.08	0.23	0.21*	0.05	
Sex (0 = male, 1 = female)	1.90	3.47	0.04	0.03	<0.01	
Income	0.41	0.93	0.04	0.07	<0.01	
Discrimination (EDS)	5.19*	2.15	0.20	0.17*	0.04	
Identification with NA Culture (NAAS)	-4.71	3.41	-0.12	-0.01	0.01	
Discrimination x Identification with NA Culture	5.37	4.23	0.10	0.09	0.01	0.01

*p<.05 (2-tailed).

### 3.3 Predicting situational pain catastrophizing

**Figure 2:**
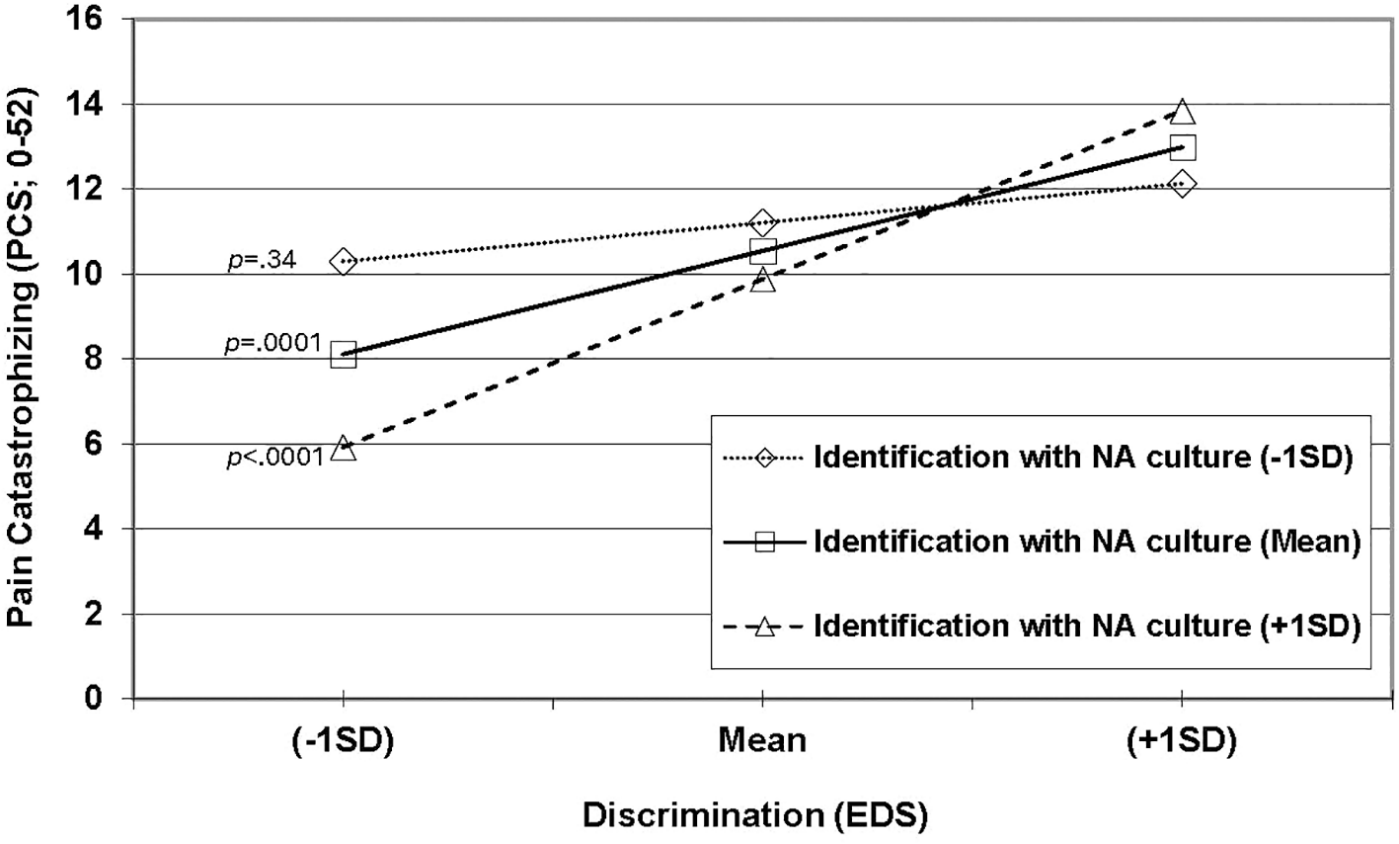
Significant interaction between identification with NA culture and interpersonal discrimination in the prediction of pain catastrophizing.

The overall model that included all predictors in the prediction of situational pain catastrophizing was significant [F(6,146) = 4.01, *p* = 0.001] and explained 14.14% of the variance. As shown in **Table 5**, a significant interaction (*p* = 0.016) was found between interpersonal discrimination (higher scores=more frequent unfair treatment) and identification with NA culture (higher scores=greater enculturation) that explained an additional 3.5% of the variance (ΔR^2^  = 0.035). As shown in **Figure 2**, the simple effect for the relationship between interpersonal discrimination and situational pain catastrophizing was significant at +1SD of identification with NA culture (*p* < 0.0001) and with the mean of identification with NA culture (*p* = 0.0001), but not at -1SD (*p* = 0.34). No other predictor in the model was significant. Taken together, these results suggest greater identification with NA culture was associated with a stronger positive relationship between interpersonal discrimination and situational pain catastrophizing.

**Table 5:** Moderated regression analysis predicting pain catastrophizing.

Predictor variable	*B*	*SEB*	β	*r*	*sr^2^*	Δ*R^2^*
Age (years)	4.64	3.95	0.10	0.09	0.01	
Sex (0 = male, 1 = female)	0.65	1.24	0.04	0.01	<0.01	
Income	0.42	0.33	0.10	0.09	0.01	
Discrimination (EDS)	3.01*	0.77	0.31	0.30*	0.09	
Identification with NA Culture (NAAS)	-1.26	1.21	-0.09	0.01	0.01	
Discrimination x Identification with NA Culture	3.67*	1.51	0.19	0.18*	0.03	0.035*

*p<.05 (2-tailed).

### 3.4 Relationship between interpersonal discrimination and identification with NA culture

Given that the moderating effect of identification with NA culture (higher scores=greater enculturation) was in the opposite direction as predicted, we examined whether identification with NA culture was associated with interpersonal discrimination (higher scores=more frequent unfair treatment) to help interpret the results. The model was significant (F[1,151] = 6.69, *p* = 0.011) and explained 4.2% of the variance. There was a significant positive relationship between identification with NA culture and interpersonal discrimination (β = 0.21), which suggests that greater identification with NA culture was associated with greater exposure to interpersonal discrimination.

## 4 Discussion

This study investigated whether identification with NA culture buffered against the harmful effects of interpersonal discrimination in a sample of healthy, pain free NA individuals. Interpersonal discrimination was positively associated with pain-related anxiety; however, this relationship was not moderated by identification with NA culture. By contrast, and surprisingly, greater identification with NA culture strengthened the relationship between interpersonal discrimination and pain catastrophizing. Additionally, greater identification with NA culture was associated with more reported experiences of interpersonal discrimination. Taken together, this highlights the ongoing devastating impact of experiencing interpersonal discrimination for NAs. These findings are discussed in the following sections.

### 4.1 Pain-related anxiety

The hypothesis that identification with NA culture would moderate (buffer) the relationship between interpersonal discrimination and pain-related anxiety was not supported. Although surprising, research has found some mixed results for the protective effects of identification with traditional culture. For example, greater participation in traditional practices buffers against the impact of discrimination on depressive symptoms for NA adults (9). Moreover, stronger identification with traditional Indigenous culture promotes happiness and is associated with less perceived stress for Yup’ik community members (39). This extends to cultural identification for other minoritized individuals too. For example, Cariello and colleagues found greater cultural identification buffered the indirect effect of anxiety through minority stressors (e.g., acculturative stress and discrimination) on physical health for Latinx immigrants (40). Conversely, Gonzalez and colleagues found that greater Indigenous cultural identification was associated with greater anxiety in Indigenous communities (41). Additionally, greater Latinx cultural identification has been found to be associated with a stronger relationship between experienced racism and anxiety for Latinx university students (42). However, it should be emphasized results from Latinx communities might not generalize to NA communities.

Age was positively related to pain-related anxiety and identification with NA culture. Thus, age could moderate the relationship between identification with NA culture and pain-related anxiety. We conducted an exploratory analysis to investigate this potential interaction (not presented), but the interaction was non-significant.

To our knowledge, this was the first study to examine whether identification with NA culture buffers the relationship between interpersonal discrimination and pain-related anxiety. In the current study, there was not a significant interaction between interpersonal discrimination and identification with NA culture when predicting pain-related anxiety. Moreover, there was an absence of a main effect for identification with NA culture when predicting pain-related anxiety, which is consistent with a prior study that found there was no association between ethnic identity and culturally related anxiety in Navajo college students (43). Our laboratory has previously found NAs experience greater pain-related anxiety than NHWs in response to pain tasks (17), thus it appears that interpersonal discrimination is associated with greater pain-related anxiety, regardless of cultural identification for participants in the current study.

### 4.2 Pain catastrophizing

Unlike pain-related anxiety, identification with NA culture did moderate the relationship between interpersonal discrimination and pain catastrophizing. As the degree of identification with NA culture increased, the relationship between interpersonal discrimination and pain catastrophizing grew stronger. This finding was surprising given the original hypothesis that greater identification with NA culture would buffer against the negative impact of interpersonal discrimination. Potential explanations for this finding are presented below.

Minoritized individuals who identify more with their traditional culture may have higher group identification, resulting in a greater threat appraisal in response to interpersonal discrimination. For example, Latinx students who had a greater degree of group identification appraised prejudice against other Latinx students as more personally threatening than participants with lower group identification (44). In turn, this led to greater depressed mood. Thus, NA individuals in the present study who reported greater identification with NA culture may have appraised experiences of interpersonal discrimination as more threatening towards their community and self-concept, thus leading to greater pain catastrophizing.

Acculturative stress could be another factor that contributes to the stronger relationship between interpersonal discrimination and pain catastrophizing in those reporting higher cultural identification. In a study with Indigenous Alaskans, it was found that discrimination was associated with higher Indigenous identification which was in turn associated with higher acculturative stress. However, this only held true for participants who reported higher identification with mainstream American culture. The authors argued that identity conflict stemming from the combined identification with American culture and Indigenous culture promoted the relationships between discrimination, Indigenous identification, and acculturative stress (45). So, given that NA participants in our sample who reported greater identification with NA culture also scored in the bicultural range of the NAAS, they may experience greater acculturative stress when experiencing interpersonal discrimination thus leading to greater pain catastrophizing. However, this hypothesis requires testing since we did not measure acculturative stress.

Another explanation might involve historical loss thinking. Tucker and colleagues (32) found that identification with NA culture and interpersonal discrimination were positively associated with historical loss thinking, which is associated with psychological distress (25, 32, 46). So, it might be that participants in the current study who identified more with NA culture engage in more historical loss thinking, creating an indirect pathway to increased pain catastrophizing. Future studies are needed to examine these potential explanations.

### 4.3 Limitations and future directions

The current study has several limitations. First, it used a cross-sectional design, thus analyses cannot determine causality or verify the temporal relationships of the effects. Second, only healthy, pain-free NA individuals were recruited because our goal was to identify variables that promote future chronic pain, so the relationships among the variables may not generalize to those experiencing chronic pain. Third, NA participants were predominately from tribal communities in Oklahoma, so results may not generalize to other NA communities. Fourth, we verified NA status from CDIB/tribal membership cards which excluded individuals who identify as NA but do not have these forms of identification. Fifth, given a majority of our sample scored below a 3 on the reverse scored NAAS (suggesting most were acculturated or bicultural in their NA cultural identification), we may not have been able to observe the protective effects of greater identification with NA culture. For example, Whitbeck and colleagues found that consistently engaging in NA traditional practices buffered the relationship between experienced discrimination and depressive symptoms (9). Thus, NA individuals who identify more with NA culture (scoring closer to a 5 on the reverse scored NAAS) may experience a greater buffering effect.

And finally, the NAAS largely focuses on acculturation processes associated with identification with NA culture. However, the NAAS does not assess for all NA spiritual beliefs/values that encompass identification with NA culture such as the interconnectedness of all things or expressing gratitude (47). Therefore, we might have missed other important facets of NA culture that buffer against the harmful effects of discrimination. Future studies should assess whether broader cultural processes like cultural connectedness or cultural continuity that include other facets such as other NA spiritual beliefs/values, engagement with elders, and intergenerational transmission of cultural values buffer against interpersonal discrimination. Cultural connectedness is defined as the degree of integration an individual has within their culture (48), whereas cultural continuity is defined as maintenance of intergenerational cultural connectedness through familial unity and elders who pass down cultural knowledge to younger generations (49). The maintenance of strong cultural connectedness by NA caregivers buffers the relationship between stressful life events and psychological distress for their children (50). Additionally, greater cultural continuity buffers the association between racial discrimination and allostatic load for Indigenous university students in Canada (51).

These findings have salient considerations for identifying cultural processes that could buffer against chronic pain risk factors. Specifically, our laboratory has found stress from interpersonal discrimination and allostatic load were both involved in indirect pathways predicting chronic pain development for NAs (4). Thus, future research should use measures that include facets of cultural connectedness and cultural continuity such as the Cultural Connectedness Scale (48) or the Native American Spirituality Scale (47) to investigate the potential buffering impact these cultural processes might have on chronic pain risk factors.

The potential mediating/moderating impact of historical loss thinking between interpersonal discrimination and chronic pain risk factors should also be explored in future studies. Interpersonal discrimination is positively associated with historical loss thinking and psychological distress (25, 32). Given that NA participants who identified more with NA culture report more interpersonal discrimination, historical loss thinking could also contribute to chronic pain risk. Lastly, acculturative stress could be explored regarding its impact on the relationship between interpersonal discrimination and chronic pain risk factors for NA individuals, especially those reporting bicultural identity (45).

## 5 Summary

This study examined identification with NA culture as a moderator between interpersonal discrimination and pain-related anxiety and pain catastrophizing among healthy, pain-free NA participants. Interpersonal discrimination was positively associated with pain-related anxiety and pain catastrophizing. Surprisingly, greater identification with NA culture was associated with a stronger relationship between interpersonal discrimination and pain catastrophizing. But we also noted that individuals who identified more with NA culture reported more experiences of interpersonal discrimination. This could explain why there was a stronger relationship between interpersonal discrimination and pain catastrophizing. To promote a strengths-based approach, other facets of identification with NA culture (e.g., cultural connectedness, cultural continuity) should be explored in future studies to identify cultural processes that protect against chronic pain risk.

## Data Availability

The raw data supporting the conclusions of this article will be made available by the authors, without undue reservation.
